# Unveiling the Upper-Extremity Morbidities of Utility-Terrain Vehicles in Pediatric Riders

**DOI:** 10.1016/j.jhsg.2024.10.005

**Published:** 2024-12-06

**Authors:** Emily M. Graham, Hunter Frederiksen, Stanley Memmott, Dana Rioux-Forker, Angela A. Wang, Douglas T. Hutchinson, Shaun D. Mendenhall

**Affiliations:** ∗ Section of Plastic and Reconstructive Surgery, University of Michigan, Ann Arbor, MI; † Spencer Fox Eccles School of Medicine at the University of Utah, Salt Lake City, UT; ‡ Saint Luke’s Plastic Surgery Specialists, Kansas City, MO; § Division of Orthopaedic Surgery, University of Utah, Salt Lake City, UT; ‖ Intermountain Primary Children's Hospital, Salt Lake City, UT; ¶ Division of Plastic & Reconstruction Surgery, Department of Surgery, University of Utah, Salt Lake City, UT

**Keywords:** ATV, Hand trauma, Mutilating hand injury, Side-by-side, UTV

## Abstract

**Purpose:**

Off-road vehicles including all-terrain vehicles (ATVs) and utility-terrain vehicles (UTVs) are preventable sources of pediatric upper-extremity (UE) trauma. We hypothesized that UE traumas from UTV accidents in children would be associated with more mutilating hand injuries, amputations, surgeries, and longer hospital and intensive care unit (ICU) admissions compared to ATVs.

**Methods:**

Pediatric cases of UE injury because of the use of an ATV or UTV at a trauma I center were identified using International Classification of Disease 9/10 codes and EPIC Boolean logic from 2010 to 2021. Findings were analyzed with Fisher exact tests, multivariate analysis of variance, analysis of variance with post hoc analyses, and multiple linear regressions.

**Results:**

Retrospective review identified 42 patients from 5 states (ATV = 25; UTV = 17). Pediatric UTV riders had triple the amount of UE vascular compromise and sustained nearly 7-times more partial hand amputations. No significant differences in time spent in the hospital or ICU were observed based on vehicle type; however, young riders of UTVs required 1.5 additional reconstructive surgeries compared to young riders of ATVs.

**Conclusion:**

Accidents caused by UTVs often lead to devastating UE injuries in pediatric riders. Hand surgeons are in a unique position to serve as forerunners to ensure pediatric rider safety and care for the devastating traumas produced by off-road vehicle accidents.

**Type of study/level of evidence:**

Therapeutic IV.

Hand trauma is a leading cause of childhood morbidity.^1^ Trauma to either hand can be devastating as the hands participate in expression, development, and industry.^2^, ^3^, ^4^, ^5^, ^6^ Severe hand trauma occurring in childhood and adolescence may also lead to disability that extends well into adulthood. Hand surgeons and emergency physicians in all settings should be familiar with the presentations, triage algorithms, and basic management of pediatric hand trauma to mitigate the risks of amputation and deformity.^7^ Among the many sources of pediatric hand trauma are injuries resulting from off-road vehicles, including all-terrain vehicles (ATVs) and utility-terrain vehicles (UTVs). The consequences of pediatric ATV use are well-documented and are associated with mortality and devastating orthopaedic trauma.^8^, ^9^, ^10^, ^11^, ^12^ Tremendous advocacy by the American Academy of Pediatrics (AAP) and other physicians have increased awareness and legislation to limit the use of these vehicles in children and adolescents.^13^^,^^14^ However, less is known regarding the outcomes of UTV-specific accidents and how these accidents impact upper- extremity (UE) morbidity.

Many reports publish ATV and UTV outcomes together and use these terms interchangeably despite the major design differences.^9^^,^^15^, ^16^, ^17^ Although both vehicles are used in adventure sport, distinguishing between these off-road vehicles is important as the design differences impact injury patterns. UTVs are typically larger, have four wheels, a steering wheel, non-straddle seating with a seatbelt, foot controls for acceleration, a roll-over protection system (ROPS), and side-by-side passenger seating (Fig. 1).^18^^,^^19^ ATVs may have three or four wheels, straddle seating without a belt or steering wheel, and do not have a ROPS.^18^ ATVs also lack side-by-side bucket seating and foot pedals as the accelerants are located on the handlebars.^18^ At our Level I trauma hospital, we observed a rising trend of mutilating UE injuries arising from the recently popularized UTV. These UE trauma cases also appeared more severe compared to the UE trauma cases typically appreciated with ATV roll-over events (ROEs) (Fig. 2). In another report consisting primarily of adults, we compared the UE presentations, injury severities, and outcomes arising from these off-road vehicles and found that UTVs are associated with significantly more UE amputations (*P* < .001) and mutilating injuries (*P* < .001).^20^ However, it is unclear if similar findings would be appreciated in young riders.

**Figure 1:**
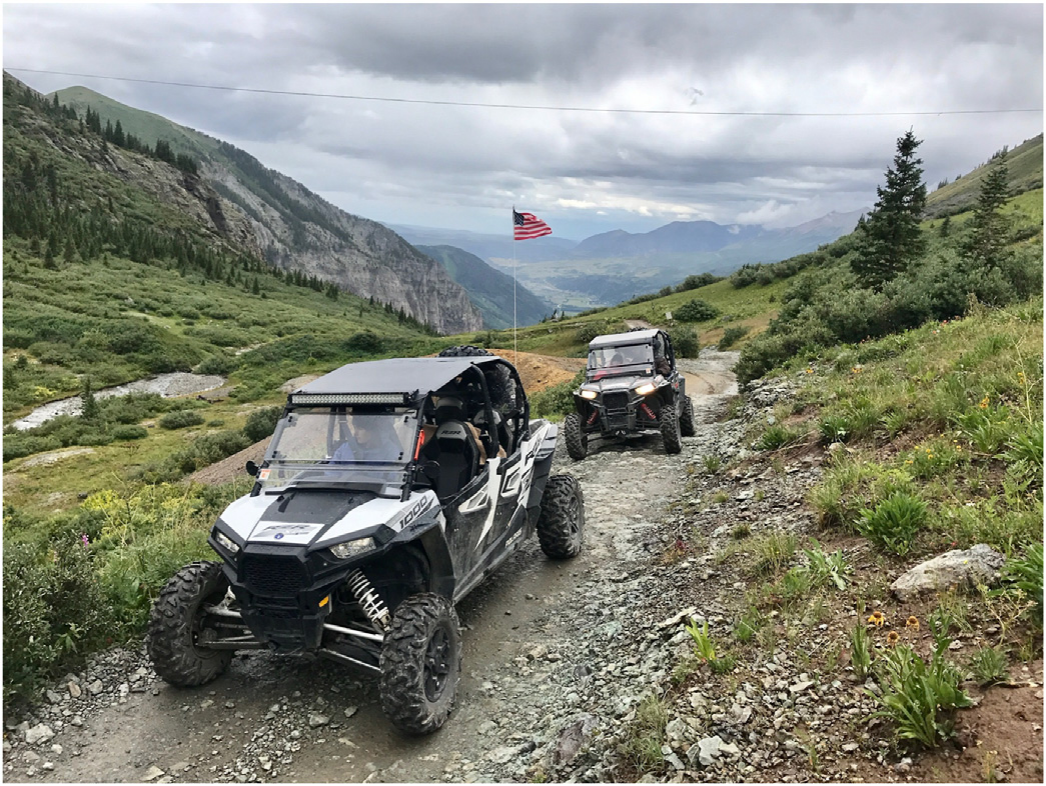
A photo of the side-by-side utility-terrain vehicle (UTV). UTVs are characterized by the presence of four wheels, a roll-over protection system over the top of the vehicle, rider seatbelts, a steering wheel, and foot pedals for accelerating. Image courtesy of Ty Call, MD.

**Figure 2:**
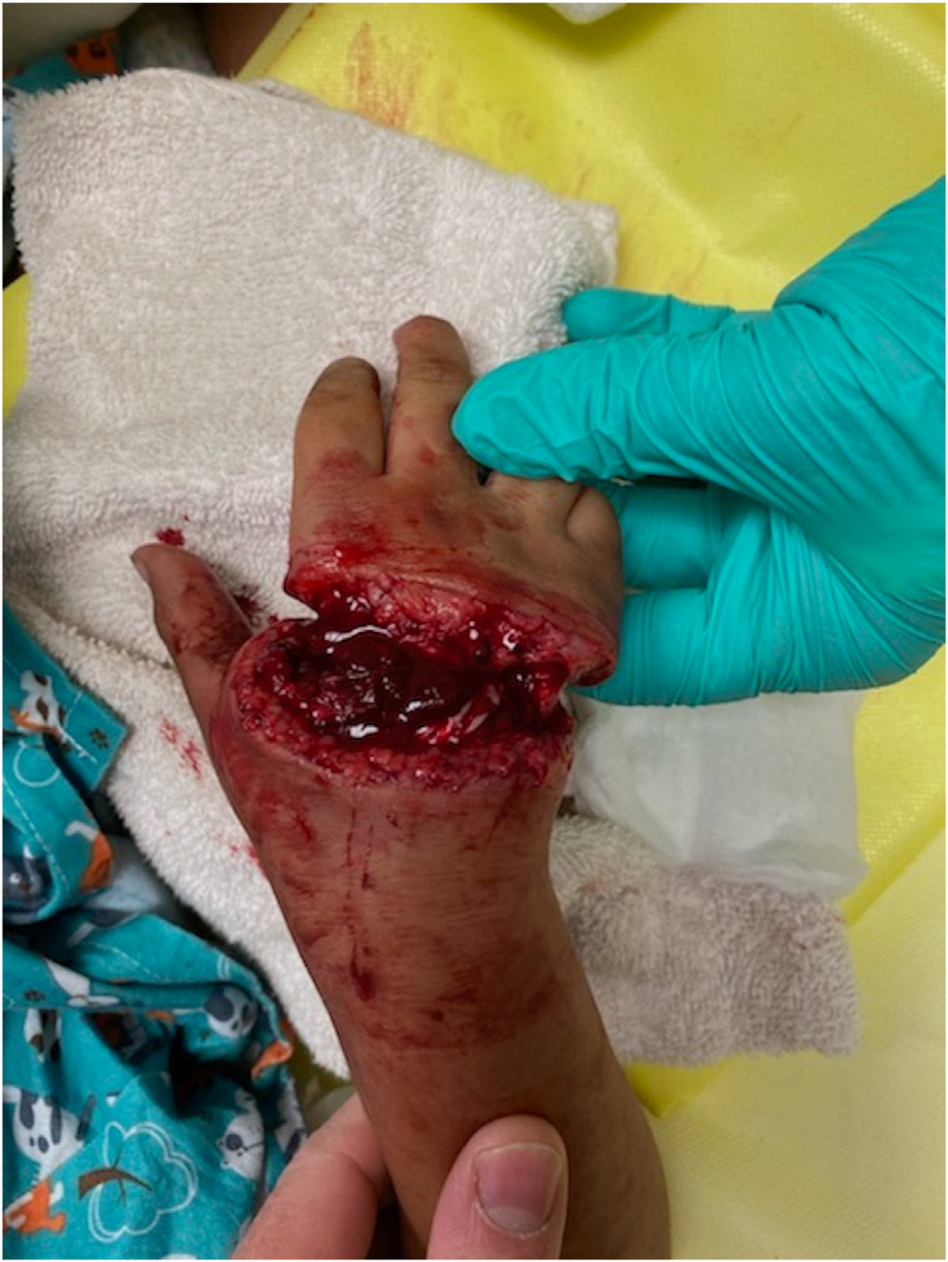
This image is a prototypical example of a thumb-sparing, transmetacarpal trauma that is commonly seen in young riders of UTV accidents. This patient was a 5-year-old girl who sustained a near partial hand amputation, with the volar skin as the only intact region following the trauma. Her operative course included fixation of metacarpals 2–5, repair of the majority of her extrinsic extensor and flexor tendons to digits 2–5, revascularization of the third and fourth common digital arteries, repair of two dorsal hand veins, and the repair of several digital nerves. Through the efforts of hand surgery and hand therapy, the need for a partial hand amputation was mitigated and the patient’s most recent DASH (Disabilities of the Arm, Shoulder, and Hand) score was 25.

The primary aim of this study was to retrospectively compare the UE morbidity arising from ATV and UTV accidents. Given the differences in vehicle design, we hypothesized that young riders in UTV accidents would present with more severe UE traumas since riders are more likely to extend their limbs outside the roll-cage zone of safety and sustain crush-avulsion injuries during a ROE compared to ATV ROE where there is no ROPS. Specifically, we hypothesized that UE traumas from UTV accidents would be associated with more mutilating hand injuries, UE amputations, UE surgeries, and longer hospital and intensive care unit (ICU) admission.

## Methods

### Search strategy and record selection

This study was approved by the Institutional Review Board at our large, Level I trauma center. A comprehensive search was conducted to identify cases occurring from January 2010 to December 2020. Patient records were identified by performing a health system data search using International Classification of Disease (ICD)-9 E821∗ and ICD-10 V86∗ codes. Medical records were also identified using Boolean logic with the health system’s WartHog search tool and the terms: “side by side,” “side-by-side,” “UTV,” “ATV,” “utility terrain vehicle,” “utility-terrain vehicle,” “utility task vehicle,” “utility-task vehicle,” “all terrain vehicle,” and “all-terrain vehicle.” Included medical records were also reviewed for vehicle descriptors, such as “steering wheel,” “foot pedals,” “roll-over,” “cage,” and “passenger seating” to ensure vehicles were accurately recorded. New cases of UE ATV and UTV injuries occurring from January 2021 to June 2021 were identified by faculty.

One of the authors performed a primary assessment of the medical records. Inclusion criteria included cases of UE injuries following ATV or UTV accidents of patients aged 0–18 years old at the time of injury. Exclusion criteria included accidents involving other off-road vehicles such as snowmobiles, off-roading trucks, and dirt bikes. Records of ATV or UTV accidents that did not involve injury to the UE were also excluded.

### Data extraction

Two independent reviewers performed a chart review of the remaining medical records. Demographic and background data included: age, gender, home location at the time of injury (state), alcohol use during the accident, helmet use, seatbelt use, Glasgow Coma Scale (GCS) at trauma bay and/or at admission, and date of injury. To assess injury severity, injury location (shoulder, arm, forearm, hand, and/or finger) and data describing the affected anatomical structures and organ systems were recorded. Counts of mutilating injuries, amputations, need for free flap coverage, and need for skin grafting were also recorded. For classification purposes, we defined mutilation as the traumatic disruption of ≥three distinct anatomic structures that invariably leads to some loss of form and function.^20^ To verify the extent of injury, operative reports, radiographs, and clinical photographs were reviewed. Vascular compromise was defined as injury to the artery and/or vein necessitating repair. Health care considerations included recording the following data: length in hospital (days), length in an ICU (days), and number of UE surgeries. The accuracy of data on the comprehensive spreadsheet was assessed independently by a third author. Differences and/or uncertainties were resolved by the senior author. Final results were reviewed as a study team.

### Statistical methods

Demographic data were reported as proportions. Comparisons between nominal variables were performed with Fisher exact test with an alpha set to 0.05. Continuous correlated dependent variables were transformed to normality and were analyzed with one-way multivariate analysis of variance (MANOVA) with separate analyses of variance (ANOVAs) for each dependent variable. ANOVAs were evaluated with an alpha of 0.025 for the Bonferroni correction. Multiple linear regressions were used to identify contributing independent variables. Regressions were interpreted with an alpha of 0.05.

## Results

### Demographics

A total of 42 cases were identified for inclusion. Of these cases, 25 were caused by ATV accidents, and 17 were caused by UTV accidents. The ATV group was predominantly women, and the UTV group was predominantly men. The ATV group included patients from four states ,and the UTV group included patients from five states. Injuries were observed in pediatric drivers and passengers. Left-sided injuries were observed in UTV drivers and in passengers directly behind the driver, and right-sided injuries were observed in UTV passengers on the contralateral side to the driver. ATV riders had a mixture of right, left, and bilateral UE traumas, regardless of seating position. Most patients in the ATV and UTV groups had a GCS of 15 and a patent airway during primary assessment. Only two, mild lower-extremity traumas were reported (ATV = 1; UTV = 1), which included subjective knee pain in one rider and a mild skin abrasion of the knee in another rider. The cases of lower-extremity traumas had normal physical examination findings and did not require additional work up or treatment. Most riders in the UTV group did not wear a seatbelt. No differences in age (*P* = .28), gender (*P* = .10), home state (*P* = .23), alcohol use (*P* = .18), helmet use (*P* = .73), airway status (*P* = .65), or GCS (*P* = .98) were observed between groups. Table 1 contains a comprehensive overview of patient demographics.

**Table 1:** Summary of Patient Demographics

	ATV		UTV			ATV		UTV	
	n	%	n	%		n	%	n	%
No. of Cases	25	59.5	17	40.5					
Age					Alcohol Use				
Range (y)	4 - 18	-	7-18		Yes	1	4.0	2	11.8
Median (y)	13	-	14		No	18	72.0	12	70.6
Gender					Unsure	7	28.0	3	17.6
M	10	40.0	11	64.7	Helmet Use				
F	15	60.0	5	29.4	Yes	2	8.0	2	11.8
FtM transgender	0	0	1	5.9	No	4	16.0	4	23.5
Home state					Unsure	19	76.0	11	64.7
Utah	21	84.0	10	58.8	Seatbelt Use				
Nevada	0	0	2	11.8	Yes	-	-	4	23.5
Idaho	1	4.0	2	11.8	No	-	-	10	58.8
Wyoming	2	8.0	1	5.9	Unsure	-	-	3	17.6
Montana	1	4.0	2	11.8	GCS				
Left-sided injuries					13–15	24	96.0	16	94.1
Driver	7	28.0	8	47.1	9–12	0	0	0	0
Passenger	1	4.0	0	0	3–8	1	4.0	1	5.9
Right-Sided injuries					Airway Status				
Driver	9	36.0	0	0	Patent	24	96.0	16	9.4
Passenger	3	12.0	9	52.9	Intubated	1	4.0	1	5.9
Bilateral injuries									
Driver	3	12.0	0	0					
Passenger	2	8.0	0	0					

FtM, female-to-male; GCS, Glasgow coma scale.

### Injury severity

**Figure 3:**
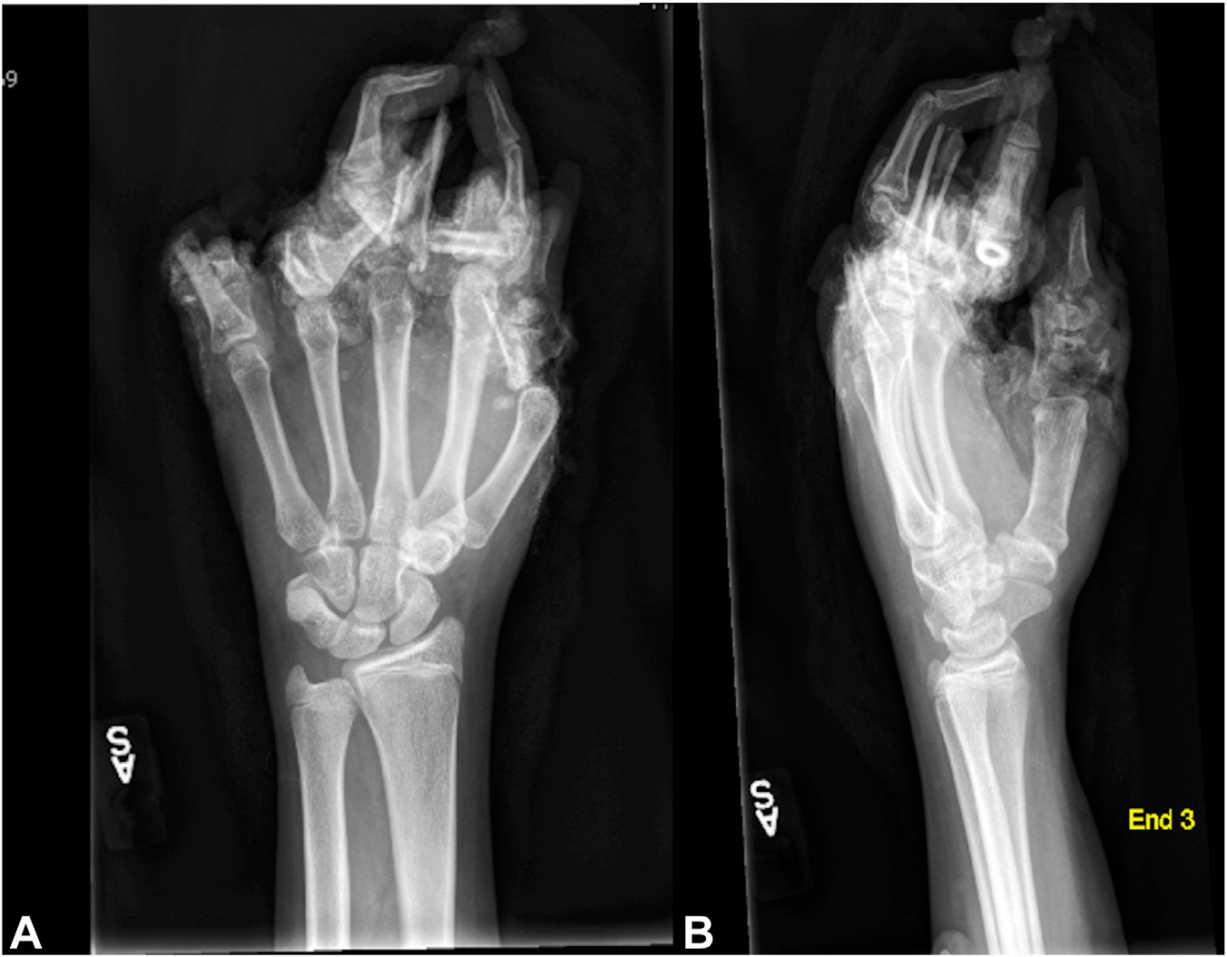
These radiographs are of a 12-year-old girl who sustained multiple open fractures after a UTV ROE. The patient was riding passenger, directly behind the driver in a four-seater UTV. During the ROE, the patient stuck her hand outside the vehicle to brace for impact. Amputations to the thumb, index, middle, and small fingers were performed as replantation was unfeasible due the extensive crush-avulsion trauma to the soft tissues.

The rates of shoulder (*P* = .65), arm (*P* = .08), elbow (*P* = .37), forearm (*P* = .08), wrist (*P* = .53), hand (*P* = .31), and finger injuries (*P* = .40) did not differ significantly between ATV and UTV groups. High rates of fractures were observed in the ATV group (20/25, 80%) and in the UTV group (14/17, 82.3%), and the rates of closed and open fractures did not vary significantly (*P* = 10 and *P* = .17, respectively) (Fig. 3). No significant difference was observed in the rate of mutilating hand injuries (*P* = .15). However, the UTV group had significantly higher rates of vascular compromise (*P* = .005) and UE amputations (*P* = .007). Of the five amputations, the first patient sustained an amputation through the small finger (SF) distal interphalangeal (DIP) joint. The second patient sustained amputations to the index finger (IF), middle finger (MF), and ring finger (RF) through the metacarpophalangeal (MCP) joints, and SF through the proximal phalanx. The third patient sustained amputations of the IF, MF, RF, and SF through the MCP joints. The fourth patient sustained amputations of the MF and RF at the proximal interphalangeal (PIP) joints. The fifth patient sustained amputations through the MF, RF, and SF through the proximal phalanges. Most of the amputations occurred distal to the MCP joint and involved more than one finger. Seatbelt use was not associated with UE amputation in UTV riders (*P* = .36). There was no difference in the rate of free flap coverage (*P* = .41), though the UTV group required significantly more skin grafts (*P* = .01). Details regarding injury severity can be viewed in Table 2.

**Table 2:** Comparison of Injury Severity by Vehicle Type

	ATV		UTV		*P*
	n	(%)	n	(%)	
Region affected					
Shoulder	2	8.0	1	5.9	.65
Arm	2	8.0	5	29.4	.08
Elbow	5	20.0	5	29.4	.37
Forearm	3	12.0	6	35.3	.08
Wrist	4	16.0	2	11.8	.53
Hand	12	48.0	6	35.3	.31
Finger	11	44.0	9	52.9	.40
Fracture					
Closed	15	60.0	6	35.3	.10
Open	5	20.0	8	47.1	.07
Mutilating injuries	8	32.0	9	52.9	.15
UE amputations	0	0	5	29.4	∗.007
Vascular compromise	4	16.0	10	58.8	∗.005
Free flap coverage	0	0	1	5.9	.41
Skin graft	3	12.0	8	47.1	∗.01

∗ Statistically significant, *P* < .05.

### Health care considerations

**Figure 4:**
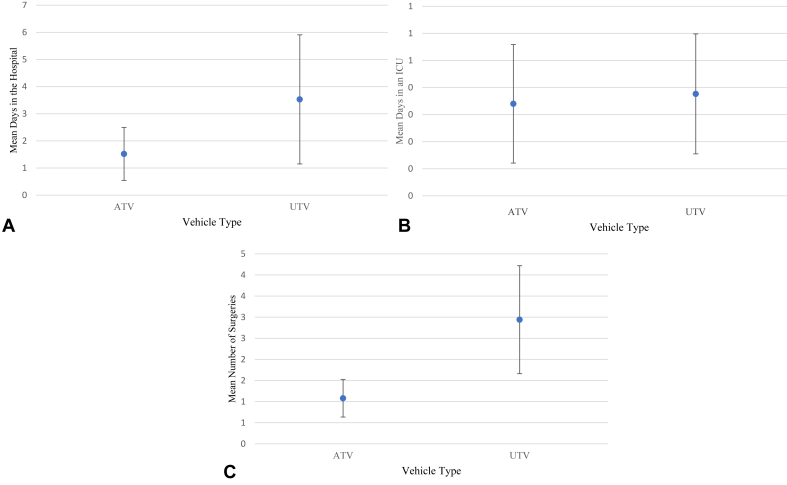
A comparison by vehicle type. **A** Average days spent in the hospital. **B** Average days spent in the ICU. **C** Number of upper extremity surgeries.

Significant differences between the ATV and UTV groups were observed with the MANOVA (*P* = .02). Days spent in the hospital were not significantly different between groups (ATV = 1.3 days; UTV = 3.1 days, *P* = .24). No significant differences were observed for days spent in the ICU (ATV = 0.61 days; UTV = 1.0 days, *P* = .46). The UTV group required significantly more UE surgeries (ATV = 1.2 surgeries; UTV = 2.7 surgeries, *P* = .003). On regression analysis, vehicle type was the only variable significantly correlated with days in the hospital (*P* = .05) and with the number of surgeries (*P* = .002) when compared with other independent variables (gender, age, helmet use, and seatbelt use). No significant correlations for days in the ICU were observed (*P* = .28) (Fig. 4).

## Discussion

An association between orthopaedic trauma and pediatric off-road vehicle use exists.^12^^,^^15^^,^^16^^,^^21^^,^^22^ Risks for severe morbidity and mortality after ATV and UTV use in children under 16 years of age prompted updated policy statements from the AAP in 2000, which discourages the use of these vehicles in all young riders.^13^ This study affirms continued effort is needed.^16^^,^^23^^,^^24^ Young riders of UTVs had triple the amount of vascular compromise and nearly seven times more partial hand amputations compared to young riders of ATVs. These findings are likely explainable by the differences in vehicle design. Young riders who extend their arms outside the UTV roll-cage zone of safety, either on purpose or accidentally during a ROE, have a high risk for sustaining severe UE trauma. Holding onto the outside ROPS or the door during a ROE may result in the extremity being pinned between the moving UTV and the ground.^25^ This mechanism of injury explains injury laterality and may contribute to the high rates of crush-avulsion injuries and vascular compromise. Because the amputations occurred distal to the MCP joint and involved more than one finger, we also infer those young riders are holding onto the outside ROPS or door during ROEs. This mechanism of injury also explain why other severe orthopaedic traumas were not appreciated in this cohort. Preventing UE morbidity from UTV accidents will require a combination of design modifications from UTV manufacturers, increased rider awareness of the associated risks, and continued physician advocacy.

**Figure 5:**
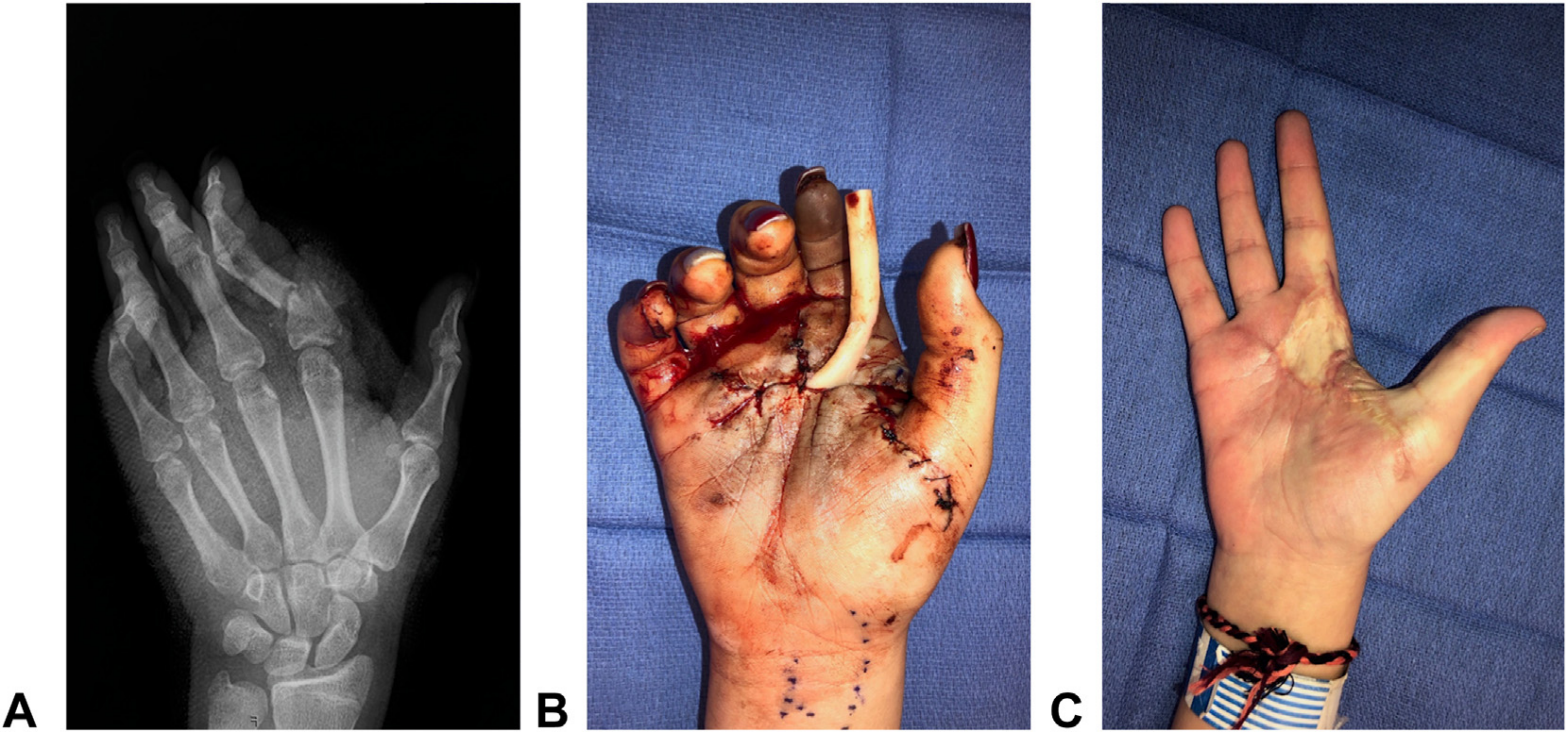
This patient was a 13-year-old girl who sustained a crush-avulsion injury to her right index finger following a utility-terrain vehicle roll-over event. The patient was initially treated at an outside hospital that was not a part of the American Society for Surgery of the Hand/American College of Surgeons (ASSH/ACS) National Hand Trauma Center Network. Image A depicts the hand at presentation to our ASSH/ACS Hand Trauma Center 24 hours after injury, with vascular compromise evident in the right index finger. The delay in appropriate microvascular treatment contributed to the need for amputation.

**Figure 6:**
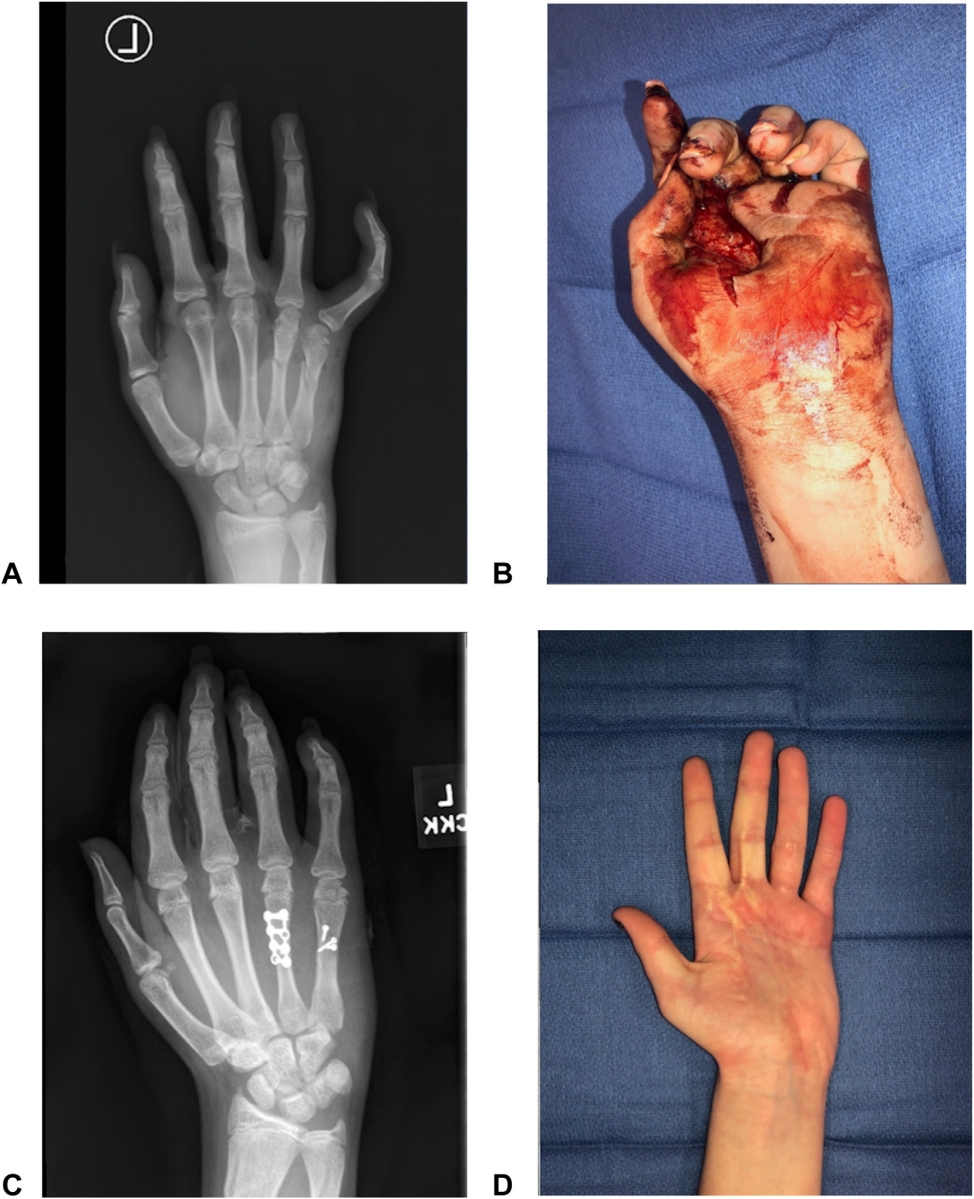
This patient was a 13-year-old girl who sustained a degloving, lacerating trauma to her left forearm and hand following a utility-terrain vehicle roll-over event. At presentation to our Hand Trauma Center, she had multiple metacarpal fractures, greenstick fractures of the distal radius and ulna, and a had a large portion of the palmar skin degloved (Image A and Image B). Bony and soft tissue traumas were addressed in a single operation (Image C). Image D shows the patient three months after her initial reconstruction. A subsequent Z-plasty was performed to release the scar contracture. At final follow-up 21 months after surgery, the patient was doing well without ongoing concerns.

The American Society for Surgery of the Hand (ASSH) recently partnered with the American College of Surgery (ACS) and the American College of Emergency Physicians to create a National Hand Trauma Center Network that contains a compilation of hospitals whose physicians provide 24/7/365 care for hand trauma emergencies.^26^ Emergency physicians and hand surgeons alike should be familiar with the nearest ASSH/ACS Hand Center as the density of these sites vary geographically.^26^ These centers are best equipped to manage all aspects of these complex UE traumas.^27^ Transport to a Hand Trauma Center with access to microsurgery and orthopaedic trauma services should be prioritized if a young rider presents with an UE amputation or near amputation from a UTV accident.^28^^,^^29^ Transport to these centers should not be delayed, even if the trauma occurred several hours prior to presentation in the emergency department (Figs. 5 and 6). Historic dogma dictated that finger and hand replantation was a viable option if the warm ischemia time was less than 6 hours and/or if the cold ischemia time was less than 12 hours.^30^ However, many of these reports were based on anectodical evidence. Current, evidence-based reports demonstrate favorable replantation outcomes even when ischemia time extends beyond the traditional window of viability.^30^, ^31^, ^32^, ^33^ Access to ACS/ASSH center resources and adequate preservation of the amputated portions are crucial for replant success. Front-line health care physicians and hand surgeons are key to ensuring these patients are transferred to the appropriate departments and centers.

Time spent in the hospital and ICU were similar as both vehicles caused mutilating injuries and presented with similar GCSs at presentation.^34^ However, young riders involved in UTV accidents required an additional 1.5 surgeries. This further attests to the increased injury severity associated with UTV accidents. Even one trauma surgery can produce negative short- and long-term psychological sequelae.^35^^,^^36^ Multiple surgeries following a severe trauma likely increase the risk of developing of post-traumatic stress disorder and anxiety. Additional medical attention is also disruptive to home, school, and work routines that amplify the emotional and financial stress of sustaining an UE injury.^37^, ^38^, ^39^ The costs of surgery also need to be considered with caretaker time off from work.^40^ If clinician advocacy is insufficient, perhaps the financial burdens associated UTV accidents will motivate policy makers, health care payers, and manufacturers to increase the safety measures of these vehicles.

In addition to supporting AAP recommendations, we also encourage partnering with manufacturers to improve vehicle safety.^14^ For example, many UTV manufacturers sell optional mesh doors, mesh windows, and wrist straps to discourage riders from extending their limbs beyond the ROPS. Making these features a permanent part of the vehicle design will improve vehicle safety. Other areas for improvement include rider education initiatives.^23^^,^^24^ As evident in this report, many pediatric riders omitted the use of helmets and seatbelts. Adherence to these safety measures should always be encouraged, regardless of rider age. Before each ride, all riders should also rehearse grabbing the seatbelt harness or dash bar to ensure their limbs stay within the vehicle during a potential ROE. Children frequently need multiple, guided rehearsals to develop kinesthetic memory. Final safety tips include minimizing reckless driving. Driver and passenger alcohol and recreational drug use should be prohibited prior to and during rides. Taking unnecessary risks and racing in unfamiliar terrains should also be discouraged. Providing short educational videos at the time of purchase and increasing safety warnings inside the vehicle will encourage young riders and their parents to adopt these safe rider habits. Increasing public awareness will likely be an important first step toward preventing severe limb trauma in ATV and UTV riders of all ages.

Our study is bound by the same limitations as other retrospective studies including selection and information bias. The lack of specific ICD-9 and ICD-10 diagnosis codes and the frequent misuse of ATV and UTV terminology was a challenge in identifying cases.^41^ The chart review process was conducted to minimize these biases, and extensive time was spent searching for UTV-specific words such as “roll-cage,” “steering wheel,” and “seatbelt” to ensure cases were accurately coded. However, there are likely cases that were not identified despite team efforts.

UTVs are a distinct class of off-road vehicles that differ from ATVs. Hand surgeons are uniquely positioned to advocate for young rider safety and increase public awareness of these devastating injuries. Educating our primary care and rural hand surgery colleagues on the unique injury patterns, severity, and UE outcomes may improve patient care. We encourage timely transfers to ASSH/ACS Hand Trauma Centers to mitigate the risks of amputation and lifelong deformity.

## Conflicts of Interest

No benefits in any form have been received or will be received related directly to this article. Dr. Shaun Mendenhall is a consultant for PolyNovo which is unrelated to this article.

## References

[1] Liu E.H., Alqahtani S., Alsaaran R.N., Ho E.S., Zuker R.M., Borschel G.H. (2014). A prospective study of pediatric hand fractures and review of the literature. Pediatr Emerg Care.

[2] Alpenfels E.J. (1955). The anthropology and social significance of the human hand. Artif Limbs.

[3] Meng S., Oi M., Saito G., Saito H. (2017). The neural correlates of biomechanical constraints in hand laterality judgment task performed from other person's perspective: A near-infrared spectroscopy study. PLoS One.

[4] Choudhury S., Blakemore S.-J., Charman T. (2006). Social cognitive development during adolescence. Soc Cogn Affect Neurosci.

[5] Kreutz-Rodrigues L., Gibreel W., Moran S.L., Carlsen B.T., Bakri K. (2022). Frequency, pattern, and treatment of hand fractures in children and adolescents: a 27-year review of 4356 pediatric hand fractures. Hand (N Y).

[6] Fetter-Zarzeka A., Joseph M.M. (2002). Hand and fingertip injuries in children. Pediatr Emerg Care.

[7] Bhende M.S., Dandrea L.A., Davis H.W. (1993). Hand injuries in children presenting to a pediatric emergency department. Ann Emerg Med.

[8] Richards J.A., Loder R.T. (2019). All-terrain vehicle use related fracture rates, patterns, and associations from 2002 to 2015 in the USA. Injury.

[9] Shults R.A., West B.A., Rudd R.A., Helmkamp J.C. (2013). All-terrain vehicle-related nonfatal injuries among young riders in the United States, 2001-2010. Pediatrics.

[10] Adil M.T., Konstantinou C., Porter D.J., Dolan S. (2017). All-terrain vehicle (ATV) injuries - an institutional review over 6 years. Ulster Med J.

[11] Denning G., Jennissen C., Harland K., Ellis D., Buresh C. (2013). All-terrain vehicles (ATVs) on the road: a serious traffic safety and public health concern. Traffic Inj Prev.

[12] Reid C.M., Rivera-Barrios A., Tapp M., Hassanein A.H., Herrera F.A. (2018). Upper extremity injuries associated with all terrain vehicle accidents: A multicenter experience and case review. Injury.

[13] (2000). All-terrain vehicle injury prevention: two-, three-, and four-wheeled unlicensed motor vehicles. Pediatrics.

[14] Flaherty M.R., Raybould T., Kelleher C.M. (2017). Age legislation and off-road vehicle injuries in children. Pediatrics.

[15] Garay M., Hess J., Armstrong D., Hennrikus W. (2017). Pediatric ATV injuries in a statewide sample: 2004 to 2014. Pediatrics.

[16] Gittelman M.A., Pomerantz W.J., Groner J.I., Smith G.A. (2006). Pediatric all-terrain vehicle-related injuries in Ohio from 1995 to 2001: using the injury severity score to determine whether helmets are a solution. Pediatrics.

[17] Shah C.C., Ramakrishnaiah R.H., Bhutta S.T., Parnell-Beasley D.N., Greenberg B.S. (2009). Imaging findings in 512 children following all-terrain vehicle injuries. Pediatr Radiol.

[18] Randall M.A. (Somewhat brief) history of the UTV industry. In *SuperATV*. https://www.superatv.com/offroad-atlas/a-somewhat-brief-history-of-the-utv-industry.

[19] Baker J. Taking the world by storm: a brief history of UTVs and their history. In *Rocky Mountain Rider Exchange*. https://www.rockymountainatvmc.com/rm-rider-exchange/taking-world-storm-brief-utv-history/.

[20] Mendenhall S.D.G.E., Memmott S., Frederiksen H., Rioux-Forker D., Wang A.A., Hutchinson D.T. (2023). A new mechanism of mutilating hand injuries: the side-by-side utility terrain vehicle. Plast Reconstr Surg.

[21] Linnaus M.E., Ragar R.L., Garvey E.M., Fraser J.D. (2017). Injuries and outcomes associated with recreational vehicle accidents in pediatric trauma. J Pediatr Surg.

[22] Rodgers G.B., Adler P. (2001). Risk factors for all-terrain vehicle injuries: a national case-control study. Am J Epidemiol.

[23] Williams R.S., Graham J., Helmkamp J.C., Dick R., Thompson T., Aitken M.E. (2011). A trial of an all-terrain vehicle safety education video in a community-based hunter education program. J Rural Health.

[24] Burgus S.K., Madsen M.D., Sanderson W.T., Rautiainen R.H. (2009). Youths operating all-terrain vehicles--implications for safety education. J Agromedicine.

[25] Charters A.C., Davis J.W. (1978). The roll-bar hand. J Trauma.

[26] American Society for Surgery of the Hand ACoS, and the American College of Emergency Physicians ASSH/ACS National Hand Trauma Center Network. https://elsevier.proofcentral.com/en-us/index.html?token=3b51be49b0aa36d014d95fb4812a3e.

[27] Ono S., Chung K.C. (2019). Efficiency in digital and hand replantation. Clin Plast Surg.

[28] Brusalis C.M., Shah A.S., Luan X., Lutts M.K., Sankar W.N. (2017). A dedicated orthopaedic trauma operating room improves efficiency at a pediatric center. J Bone Joint Surg Am.

[29] Sabapathy S.R., Satbhai N.G. (2014). Microsurgery in the urgent and emergent management of the hand. Curr Rev Musculoskelet Med.

[30] Harbour P.W., Malphrus E., Zimmerman R.M., Giladi A.M. (2021). Delayed digit replantation: what is the evidence?. J Hand Surg Am.

[31] Levin L.S. (2018). Commentary on "Immediate versus overnight-delayed digital replantation: comparative retrospective cohort study of survival outcomes". J Hand Surg Am.

[32] Woo S.H., Cheon H.-J., Kim Y.-W., Kang D.-H., Nam H.-J. (2015). Delayed and suspended replantation for complete amputation of digits and hands. J Hand Surg Am.

[33] Cavadas P.C., Rubí C., Thione A., Pérez-Espadero A. (2018). Immediate versus overnight-delayed digital replantation: comparative retrospective cohort study of survival outcomes. J Hand Surg Am.

[34] Killingsworth J.B., Tilford J.M., Parker J.G., Graham J.J., Dick R.M., Aitken M.E. (2005). National hospitalization impact of pediatric all-terrain vehicle injuries. Pediatrics.

[35] Biçen Ç., Akdemir M., Gülveren D., Dirin D., Ekin A. (2021). Depression, anxiety, and post-traumatic stress disorder following orthopedic war injuries. Cureus.

[36] Ongecha-Owuor F.A., Kathuku D.M., Othieno C.J., Ndetei D.M. (2004). Post traumatic stress disorder among motor vehicle accident survivors attending the orthopaedic and trauma clinic at Kenyatta National Hospital, Nairobi. East Afr Med J.

[37] Banu T., Chowdhury T.K., Aziz T.T. (2018). Cost incurred by the family for surgery in their children: a Bangladesh perspective. World J Surg.

[38] Racimo A.R., Talathi N.S., Zelenski N.A., Wells L., Shah A.S. (2018). How much will my child's operation cost? Availability of consumer prices from US hospitals for a common pediatric orthopaedic surgical procedure. J Pediatr Orthop.

[39] Schuster M.A., Chung P.J., Elliott M.N., Garfield C.F., Vestal K.D., Klein D.J. (2009). Perceived effects of leave from work and the role of paid leave among parents of children with special health care needs. Am J Public Health.

[40] Gudnadottir G., Tennvall G.R., Stalfors J., Hellgren J. (2017). Indirect costs related to caregivers' absence from work after paediatric tonsil surgery. Eur Arch Otorhinolaryngol.

[41] Innes K., Hooper J., Bramley M., DahDah P. (1997). Creation of a clinical classification. International statistical classification of diseases and related health problems--10th revision, Australian modification (ICD-10-AM). Health Inf Manag.

